# Type 2 diabetes and succinate: unmasking an age-old molecule

**DOI:** 10.1007/s00125-023-06063-7

**Published:** 2024-01-05

**Authors:** Sonia Fernández-Veledo, Anna Marsal-Beltran, Joan Vendrell

**Affiliations:** 1 Hospital Universitari Joan XXIII de Tarragona, Institut d’Investigació Sanitària Pere Virgili (IISPV)-CERCA, Tarragona, Spain; 2 CIBER de Diabetes y Enfermedades Metabólicas Asociadas (CIBERDEM)-Instituto de Salud Carlos III (ISCIII), Madrid, Spain; 3 Universitat Rovira I Virgili (URV), Reus, Spain

**Keywords:** Diabetic nephropathy, Diabetic retinopathy, Gestational diabetes, Microbiota, NAFLD, Review, Succinate, SUCNR1, Type 1 diabetes, Type 2 diabetes

## Abstract

Beyond their conventional roles in intracellular energy production, some traditional metabolites also function as extracellular messengers that activate cell-surface G-protein-coupled receptors (GPCRs) akin to hormones and neurotransmitters. These signalling metabolites, often derived from nutrients, the gut microbiota or the host’s intermediary metabolism, are now acknowledged as key regulators of various metabolic and immune responses. This review delves into the multi-dimensional aspects of succinate, a dual metabolite with roots in both the mitochondria and microbiome. It also connects the dots between succinate’s role in the Krebs cycle, mitochondrial respiration, and its double-edge function as a signalling transmitter within and outside the cell. We aim to provide an overview of the role of the succinate–succinate receptor 1 (SUCNR1) axis in diabetes, discussing the potential use of succinate as a biomarker and the novel prospect of targeting SUCNR1 to manage complications associated with diabetes. We further propose strategies to manipulate the succinate–SUCNR1 axis for better diabetes management; this includes pharmacological modulation of SUCNR1 and innovative approaches to manage succinate concentrations, such as succinate administration and indirect strategies, like microbiota modulation. The dual nature of succinate, both in terms of origins and roles, offers a rich landscape for understanding the intricate connections within metabolic diseases, like diabetes, and indicates promising pathways for developing new therapeutic strategies.

**Graphical Abstract:**

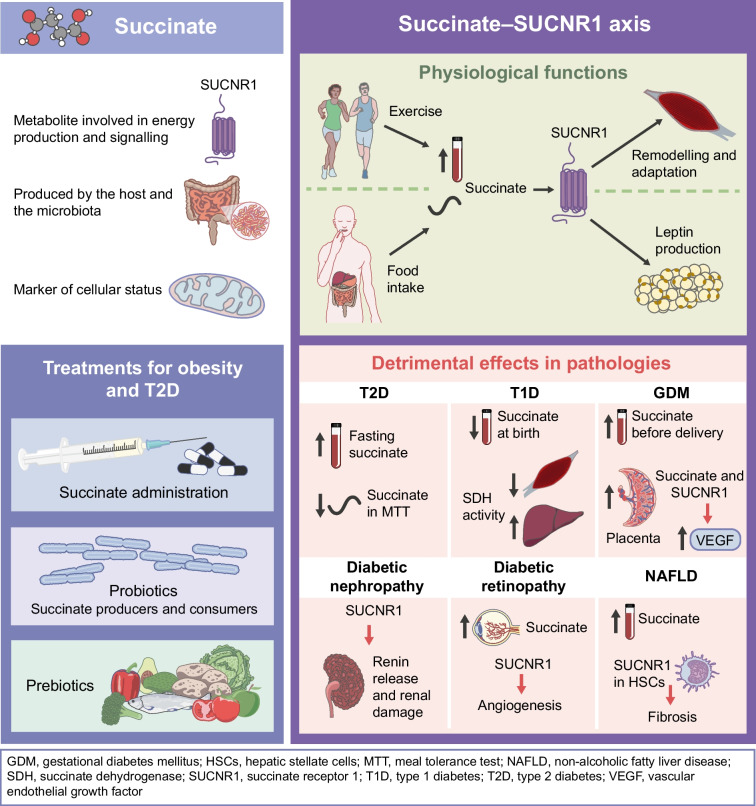

**Supplementary Information:**

The online version contains a slide of the figure for download available at 10.1007/s00125-023-06063-7.

## Succinate: a dual metabolite from multifaceted perspectives—origin and functions

Succinate, a key dicarboxylic acid in energy metabolism, has two main origins in humans: the mitochondria and the gut microbiota [1, 2]. Understanding the diverse origins of succinate provides important insights into the complex interplay between host metabolism and the gut microbiota, with potential implications for health and disease.

### Succinate: born in the mitochondria—connecting the Krebs cycle to mitochondrial respiration

**Fig. 1:**
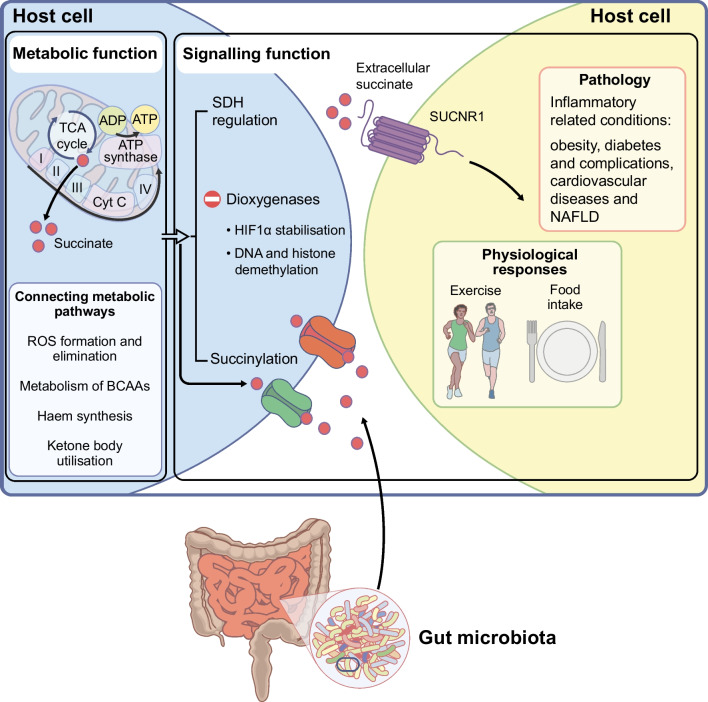
The dual nature of succinate: origins and functions. Succinate (depicted by red circles), which is generated both by the host and the microbiota, is widely recognised as a key metabolic substrate crucial for ATP production. Within the mitochondria, succinate serves as the link between the Krebs cycle (also known as the tricarboxylic acid cycle [TCA]) and respiration, influencing various pathways such as reactive oxygen species (ROS) production, branched-chain amino acid (BCAA) metabolism, haem synthesis and utilisation of ketone bodies. However, succinate’s function extends beyond these metabolic roles. It can also be translocated to the intracellular space where it plays several signalling roles, including dioxygenase inhibition (thus, promoting stabilisation of hypoxia inducible factor 1 subunit alpha [HIF1α] and DNA/histone demethylation), protein succinylation and allosteric modulation of the SDH enzyme. Additionally, succinate can be transported outside of the cell via a series of transporters, where it interacts with its specific receptor, SUCNR1. Upon this interaction, succinate functions similarly to a hormone, leading to the activation of cell-specific signalling pathways. Signal transduction associated with the succinate–SUCNR1 axis contributes to physiological responses to factors such as exercise and food intake. However, its overactivation has also been implicated in the development of metabolic disorders, including obesity, and diabetes and its related complications. I–IV, Complex I–IV; Cyt C, cytochrome *c*. This figure is available as a downloadable slide

Within mitochondria, succinate comes from converting α-ketoglutarate via a Krebs cycle enzyme, succinyl-CoA synthetase, which is central to ATP production. Succinate links the Krebs cycle to mitochondrial respiration via succinate dehydrogenase (SDH), facilitating the oxidation of succinate to fumarate and transferring electrons to the electron transport chain (ETC) for ATP generation. As a key intermediary between the Krebs cycle and mitochondrial respiration (Fig. 1), succinate has gained attention as a potential biomarker of cellular energy state. Dysregulation in cellular metabolism triggered by factors such as tissue damage, hypoxia and immune activation, can lead to alterations in intracellular succinate levels, resulting in increased circulating levels [1]. Accumulation of succinate is associated with conditions like obesity [3], diabetes [3–5], cardiovascular diseases [6–9] and non-alcoholic fatty liver disease (NAFLD) [10–12]. Thus, monitoring succinate levels in blood could help diagnose, predict risk and develop treatments for these conditions.

### Succinate: a metabolite of microbial ancestry

The gut microbiota metabolises dietary and host nutrients, producing beneficial compounds like short-chain fatty acids (SCFAs) and organic anions, including succinate. Once seen as only an SCFA precursor, recent findings highlight succinate as a byproduct of anaerobic fermentation from Bacteroidetes phylum, particularly *Bacteroides* and *Prevotella* genus, which are primary succinate producers in the mammalian gut [13]. Succinate levels in faeces are generally low due to bacterial cross-feeding, resulting in succinate being converted into propionate. Like SCFAs, microbiota-derived succinate can be an energy source for intestinal cells [14] and can regulate the intestinal immune system [15]. Microbially produced succinate can enter the bloodstream, contributing to systemic succinate levels. While mitochondrial succinate production seems to be the primary source of succinate in healthy individuals [16], dysbiosis conditions, like inflammatory bowel disease and obesity, show a clear association between gut microbiota and circulating succinate in humans [3, 15]. Notably, the gut microbiota is a major source of elevated succinate levels in obesity [16]. Hence, targeting microbial succinate production might be a promising therapeutic strategy. As described below, however, metabolic studies reveal benefits from both succinate-producing [14, 17–19] and -consuming bacteria [3, 16, 20] at the molecular level, which underscores the complex symbiosis between the host metabolism and gut microbiota.

### Succinate as a double agent: intracellular and extracellular signalling mechanisms of a versatile molecule

Succinate’s functional repertoire extends beyond the confines of the respiratory chain, positioning it as a critical metabolic crossroads. Its metabolism is entwined with diverse pathways, including homeostasis of mitochondrial reactive oxygen species (ROS), metabolism of branched-chain amino acids, haem synthesis and utilisation of ketone bodies [21]. Further, it moonlights as a signalling molecule, exerting diverse functions within and outside of the cell (Fig. 1). Intracellularly, succinate also functions as a signalling molecule in three main ways: (1) it acts as a competitive inhibitor of α-ketoglutarate-dependent dioxygenases, influencing processes like DNA and histone demethylation, hypoxic response and epigenetic regulation; (2) it serves as an allosteric modulator of metabolic enzymes, like SDH, creating a positive feedback loop; and (3) it acts as a substrate for succinyl-CoA, enabling post-translational modification of proteins via succinylation, which regulates metabolic enzyme activities [1].

Succinate’s role as an extracellular signalling molecule was unravelled with the landmark discovery of succinate receptor 1 (SUCNR1, also known as G protein-coupled receptor 91 [GPR91]) [22]. As a member of the G protein-coupled receptor (GPCR) family, SUCNR1 exhibits wide tissue distribution, being present in adipose tissue, the liver, intestine and kidney [23]. The receptor is largely specific for succinate, with other carboxylic acids showing comparatively lower binding affinities [1, 22]. The extracellular region of SUCNR1 governs ligand accessibility, while the intracellular region manages signalling transmission. SUCNR1 activation initiates interaction with heterotrimeric GTPases, thereby stimulating downstream signalling events that vary with cell type, leading to different downstream signals and effects in a cell-dependent manner. Succinate–SUCNR1 signalling has been implicated in various transduction pathways, such as ERK pathways in cardiomyocytes [24] and AMP-activated protein kinase (AMPK) pathways in adipocytes [25]. Though the desensitisation and internalisation processes of SUCNR1 are similar to those of other GPCRs, our understanding of these mechanisms remains rudimentary.

Initially known as a GPCR involved in inflammatory pathologies [4, 15, 26, 27], our understanding of SUCNR1 has evolved to consider it as a critical regulator of the complete inflammatory response, particularly in macrophages. During the early stages of inflammation, succinate is produced to elicit a robust response, but it also exerts anti-inflammatory effects, thereby participating in resolving inflammation [1, 27, 28]. However, prolonged metabolic stress, such as with obesity and type 2 diabetes, disrupts this coordinated mechanism [27], tipping the balance towards a proinflammatory response, thereby contributing to chronic inflammation [28].

Further, emerging research has challenged the conventional belief of SUCNR1 being inactive under healthy conditions. Studies have observed transiently elevated succinate levels during physiological states, like exercise [29] and food intake [30], implying additional metabolic functions. In this context, it has been revealed that SUCNR1 signalling contributes to paracrine communication in skeletal muscle during exercise, resulting in muscle remodelling [29] and controls leptin production by adipose tissue in response to food ingestion [25]. The transient increase in succinate levels, which appears to be essential for regulating physiological responses to exercise and feeding via SUCNR1, differs notably from the consequences of chronic succinate elevation that are observed in metabolic disorders. These disparities between acute increases in health and chronic rises in disease conditions parallel findings related to blood glucose and inflammation. Furthermore, chronic elevation may induce a succinate-resistant state, similar to the observed phenomenon of leptin resistance in the context of obesity, where hyperleptinaemia is associated with reduced leptin sensitivity [31]. Consequently, the effects of succinate administration may vary, proving either beneficial or detrimental depending on the specific pathophysiological state (see ‘Succinate administration’ section below for details), with potential favourability limited to the early stages of disease.

In summary, succinate is a remarkably versatile metabolite, acting as a pivotal constituent in metabolic pathways and an effector molecule that influences cell behaviour. Succinate’s multifaceted nature helps to maintain cellular homeostasis and coordinate physiological responses. Nevertheless, the chronic elevations in succinate that are observed in metabolic disorders are closely linked to disease progression. The role of succinate and SUCNR1 in diabetes will be further explored in the subsequent sections.

## Diabetes and the succinate–SUCNR1 axis

The role of succinate and its receptor in the pathophysiology of diabetes has gained significant attention in recent years. This section aims to provide a comprehensive review of the current knowledge on the interplay between diabetes and the succinate–SUCNR1 axis, shedding light on the underlying mechanisms and potential therapeutic implications.

### Succinate levels in diabetes

Precise quantification of succinate levels within the circulatory, faecal and intracellular environments is essential to fully understand the role of succinate as both a metabolic and signalling molecule in the context of diabetes.

#### Type 2 diabetes

In the context of metabolic disorders, specifically in rodent models of type 2 diabetes, obesity and hypertension, Sadagopan and colleagues were pioneers in documenting elevated levels of circulating succinate [9]. These findings generated considerable interest; however, Sadagopan et al were not able to replicate them in studies involving humans with hypertension or diabetes. Nonetheless, subsequent research unveiled that individuals with type 2 diabetes and obesity did, in fact, exhibit elevated levels of succinate in the circulation [3, 4]. The discrepancies in findings may have resulted from variations in the analytical methods used or may be owing to the differing phenotypic characteristics of the human cohorts used. Several studies show that increased succinate levels in the blood correlate with BMI, insulin, glucose, HOMA-IR and plasma triglycerides [3, 5, 16, 30], with changes in microbiota composition leading to an increased ratio of succinate producers:consumers [3]. Our recent study involving individuals with class III obesity demonstrated a negative correlation between circulating and faecal succinate [16], which hints at a possible overflow of succinate into the systemic circulation within the obesity context.

The elevated succinate levels observed in obesity and type 2 diabetes tend to decrease 1 year after metabolic surgery [5, 30], potentially due to weight loss and reduced inflammation, though these effects may vary over time. Interestingly, a bypass surgery study reported an increase in circulating succinate at 3 months post-surgery, which correlated with decreased jejunal levels [32]; this may indicate a shift in substrate flow and utilisation during the early stages after surgery. Furthermore, succinate levels are influenced by nutritional status, with changes seen in response to a mixed meal [30] typically being observed in healthy individuals. In contrast, this response was lost in individuals with obesity and type 2 diabetes, although recoverable post-surgery [30]. Interestingly, adults with the metabolic syndrome and a late chronotype displayed reduced insulin sensitivity and higher fasting succinate levels than those with an early chronotype [33]. Thus, it is reasonable to consider succinate as a potential biomarker for type 2 diabetes. In fact, our findings suggested that pre-bariatric surgery circulating succinate could indicate potential for diabetes remission and help to identify the best surgical procedure to achieve it [5].

#### Type 1 diabetes

In the context of type 1 diabetes, succinate has not been deeply explored. However, reduced serum levels at birth in children who later developed type 1 diabetes [34] suggest its potential as a predictive biomarker. In relation to succinate and type 1 diabetes, most research has investigated changes in SDH activity and mitochondrial function (these are less studied in type 2 diabetes). Consistent findings indicate a decrease in SDH activity in the muscles of rat models of diabetes [35] and people with type 1 diabetes [36]. Notably, individuals with type 1 diabetes maintain a physiological succinate response to exercise in peripheral blood [37]. These studies have mainly involved male participants, demanding further research in female participants. By contrast, increased SDH activity has been documented in the liver in type 1 diabetes [38]. The implications of these variations require further investigation.

#### Gestational diabetes

Succinate levels rise before delivery in women with gestational diabetes mellitus (GDM), particularly those receiving insulin treatment [39]. This suggests that the increase in succinate observed may primarily be due to insulin administration or GDM severity. Additionally, increased levels of succinate and SUCNR1 have been observed in placental tissue from women with GDM [40]. SUCNR1, which is present in endothelial cells, can promote proliferation, chemoattraction, wound healing and vascular endothelial growth factor (VEGF) production [40], potentially contributing to the increased angiogenic response in GDM.

### Succinate–SUCNR1 signalling in diabetes-related complications

Research highlights the association between abnormal succinate levels, hyperactivation of SUCNR1 and various disorders, including diabetes and its related complications. Maintaining a balanced succinate–SUCNR1 axis may be crucial for optimal physiological functioning, with dysregulation potentially contributing to diseases like diabetes. Indeed, higher succinate levels were noted in people with diabetic complications compared with those with well-controlled type 2 diabetes [41]. A SUCNR1 polymorphism (rs73168929), affecting the 3′ untranslated region (UTR) of the gene, which is an important zone involved in miRNA binding, has recently been linked to type 2 diabetes and hypertension susceptibility in a Chinese population [42]. Thus, changes in SUCNR1 expression due to alterations in miRNA binding may serve as a predictive biomarker of type 2 diabetes and hypertension, although further research is needed. The role of SUCNR1 activation in common diabetic complications, including diabetic nephropathy, retinopathy, and NAFLD, all of which have a significant impact on patient health, is the focus of ongoing research and the following subsections.

#### Diabetic nephropathy

Diabetic nephropathy, which affects 20–50% of individuals with diabetes, is a widespread, costly long-term diabetes complication and the main cause of end-stage renal disease. Its progression is linked to overactivation of the renin–angiotensin system, which is crucial for blood pressure regulation and renal balance. Overactivation of the renin–angiotensin system leads to renal inflammation, fibrosis, endothelial dysfunction and progressive kidney damage [43]. Succinate’s role in renin release, first identified in rat glomeruli in 1976 [44], is SUCNR1-dependent [22], corroborated by elevated receptor expression in kidney [22, 23]. High succinate levels in the urine of individuals with progressive diabetic nephropathy [45] and of murine models of streptozocin-induced type 1 diabetes [43], along with high glucose driving increased renin and prorenin release via activation of the succinate–SUCNR1 pathway [43], underscore this significance of the succinate–SUCNR1 axis in the pathophysiology of diabetic nephropathy. Expressed in glomerular endothelial cells and the macula densa, SUCNR1 influences renin production by triggering secretion of prostaglandin, cyclooxygenase (COX)-1, COX-2 and endothelial nitric oxide synthase (eNOS) [46]. SUCNR1’s expression extends to tubular cells, which are the predominant producers of (pro)renin in individuals with diabetes [47]. Furthermore, succinate enhances renal damage by inducing renal tubular cell apoptosis via the ERK pathway [48]. Despite these insights suggesting that SUCNR1 inhibition may be a possible treatment avenue for diabetic nephropathy, no such interventions have been executed.

#### Diabetic retinopathy

Diabetic retinopathy is intricately tied to SUCNR1; this receptor is found in key areas of the retina, such as the retinal ganglion cell layer and the retinal pigment epithelium [49]. Research involving *Sucnr1*-deficient mice highlights the receptor's importance in retinal development and function as these mice exhibit early sub-retinal dystrophy [49]. Studies further indicate that modulation of SUCNR1 through succinate supplementation or SUCNR1 knockdown can influence retinal vasculature development [50], thereby asserting a role for succinate-mediated SUCNR1 function in maintaining retinal vascular health. Recent investigations, however, also implicate succinate and SUCNR1 in the progression of diabetic retinopathy, particularly in retinal vascular dysfunction and neurodegeneration [51]. Observations of elevated succinate levels in the vitreous fluid of individuals with active proliferative diabetic retinopathy [52] and in retinas from rat models of diabetes [53] underscore a link between succinate accumulation, hypoxia and retinal neovascularisation, key pathological features of diabetic retinopathy [51]. Interestingly, fasting succinate levels in the serum of individuals with proliferative retinopathy exceed those of people with type 2 diabetes [54]. In contrast, individuals with diabetic retinopathy exhibit lower faecal succinate levels than healthy control participants [55], echoing the inverse relationship between circulating and faecal succinate levels that have been found in obesity studies [16]. Experimental use of *Sucnr1* short hairpin RNA (shRNA) in diabetic rats has demonstrated retinal damage reduction and functional improvement [53]. Mechanistically, succinate-mediated SUCNR1 activation has been connected to angiogenesis, wherein hyperglycaemia-induced increases in succinate levels activate SUCNR1, stimulating VEGF release and promoting endothelial cell proliferation and migration in vitro [56]. Recent work has also highlighted the interaction between iron, SUCNR1 and the renin–angiotensin system in diabetes-related neurodegeneration and vascular abnormalities [57], stressing the role of iron homeostasis in preventing retinal oxidative stress. Collectively, these findings underline the critical involvement of succinate and SUCNR1 in the pathogenesis of diabetic retinopathy, suggesting new potential therapeutic targets.

#### NAFLD

NAFLD, affecting roughly 25% of the global population, is linked with insulin resistance, obesity and type 2 diabetes, with prevalence soaring to 50–70% in people with diabetes [58]. The role of succinate and SUCNR1 in the progression of NAFLD is under investigation, especially as high blood succinate levels are found in individuals with NAFLD [10–12]. Notably, the analysis of recent clinical data has further highlighted the potential of succinate as a non-invasive biomarker for diagnosing fatty liver [12]. Research has also reported elevated SUCNR1 expression in the liver during non-alcoholic steatohepatitis (NASH) in both animal models [12, 26] and humans [12, 59], hinting that SUCNR1 expression might serve as a valuable prognostic marker for NASH [12]. Mechanistic studies in cell cultures and animal models show that hepatocyte-released succinate triggers fibrotic changes in the liver through activation of hepatic stellate cells [26, 60], suggesting the potential of SUCNR1 as a target for anti-fibrotic NAFLD treatment [61]. However, the contribution of SUCNR1 signalling in other liver cell populations to NAFLD progression remains underexplored. In fact, our latest findings propose that the succinate–SUCNR1 pathway might be protective in early NAFLD by mitigating lipid accumulation and glycogen depletion in damaged hepatocytes [12]. Further investigations are warranted, but these recent findings suggest that cell-directed pharmacology could be a more effective strategy than SUCNR1 agonists or antagonists for managing NAFLD [62].

## Strategies for manipulating the succinate–SUCNR1 axis in the management of diabetes

### Pharmacological modulation of SUCNR1: the interplay of agonists and antagonists

The succinate–SUCNR1 axis, with SUCNR1 acting as a GPCR, presents a promising target for innovative diabetes therapies. SUCNR1 exhibits broad tissue distribution in a cell-specific manner, although its expression profile may vary across species. According to data from the Human Protein Atlas (www.proteinatlas.org/search/sucnr1, accessed 9 October 2023), SUCNR1 is prominently expressed in the human kidney, thyroid gland and bone marrow. At a cellular level, it is highly expressed in proximal tubular cells, macrophages and monocytes. However, in mice, SUCNR1 is mostly located in the adipose tissue, liver and kidney [23, 63]. This variability in tissue distribution, coupled with its cell-specific functions, should be considered when contemplating SUCNR1 as a pharmacological target.

Small molecules acting as agonists or antagonists can modulate this receptor. In 2017, two SUCNR1 agonists, *cis*-epoxy succinic acid and *cis*-1,2-cyclopropane carboxylic acid, were discovered. They showed significant in vivo activity and comparable efficacy to succinate, albeit without the corresponding intracellular actions. Indeed, the former was 10- to 20-fold more potent than the natural ligand [64]. Later, novel agonists with higher stability were identified [65]. SUCNR1 antagonists, such as the human-specific NF-56-EJ40 [66], were also reported, underscoring the need for compounds with activity on rodent receptor orthologues so that the effects of SUNCR1 antagonists can be further explored using preclinical models. Notably, agonist and antagonist tracers for mouse and human SUCNR1 orthologues have recently been developed [67]. SUCNR1 antagonists offer potential benefits in counteracting the harmful effects of succinate signalling observed in diabetes, promising a reduction in inflammation and metabolic balance restoration. Inhibiting SUCNR1 could prevent diabetes-related retinal neovascularisation [50] and kidney disease [43], although these potential benefits have yet to be scientifically substantiated. However, using SUCNR1 as a pharmaceutical target requires an extensive understanding of its physiological and pathological roles. In fact, in the context of NAFLD, while SUCNR1 antagonists may reduce fibrosis [61], they could also worsen steatosis [12]. Similarly, while the inhibition of SUCNR1 has been proposed as a strategy to alleviate various inflammatory conditions [4], its blockade might be detrimental in treating inflammation and glucose intolerance in obesity [27]. However, no preclinical studies involving animal models treated with selective SUCNR1 modulators as pharmacological therapies have been reported to date.

### Strategies for manipulating succinate concentrations

Circulating levels of succinate can be influenced by cellular mitochondrial activity, microbiota composition and diet. As discussed below, several studies have explored the impact of dietary succinate supplementation and probiotic modulation on energy homeostasis, though some of the findings have been inconsistent.

#### Succinate administration

Despite chronically high succinate levels being a characteristic of metabolic diseases [3, 5, 6, 10–12], some research has examined its therapeutic use in obesity and diabetes management (Table 1). In animal models of type 1 diabetes, succinate administration has been shown to alleviate liver damage and lower lipid peroxidation [68]. Combined with oleic acid, it improves the control of blood glucose levels and promotes weight loss [69]. Research using short-term high-caloric diets in mice or genetic models of obesity has indicated that succinate can stimulate beige adipose tissue development [70], induce thermogenesis in brown adipose tissue [71] and improve glucose homeostasis [71]. Specifically, succinate was found to improve glucose homeostasis and reduce hyperglycaemia by activating intestinal gluconeogenesis [14, 18]. Most of the available research concerning the therapeutic potential of succinate has predominantly focused on obesity rather than diabetes, often emphasising a preventative rather than therapeutic strategy. It is noteworthy that the development of hyperglycaemia and impaired glucose-stimulated insulin secretion in high-fat-diet (HFD)-induced obesity models occur over a period of time, akin to the mild progression of diabetes observed in humans [72, 73]. Indeed, detrimental effects have been observed with extended treatment regimens, where succinate supplementation has been shown to increase fasting glucose and LDL-cholesterol levels without significantly affecting body weight, albeit with a reduction in adiposity [74]. These findings collectively suggest that the timing of succinate administration may hold a pivotal role in achieving favourable outcomes. Consequently, further investigations will be imperative to explore the potential of succinate supplementation once the pathology is already established. In addition, studies on zebrafish, an appealing model for obesity and type 2 diabetes, have yielded deleterious outcomes concerning weight gain, hepatic fat accumulation and gut microbiota composition [75], which points to potential species differences.

**Table 1:** Effects of succinate administration in preclinical models of diabetes and obesity

Outcome	Dose	Effects	Model used	Reference
Protective				
	Succinic acid disodium salt hexahydrate (50 mg/kg BW) by daily i.p. injection for 30 days	Decrease in liver damage and lipid peroxidation	STZ-induced diabetic male rats	[68]
	Oleic acid + succinic acid (1:1; 800 mg/kg BW) dissolved in 0.5 ml of 10% polysorbate 20, administered daily for 4 weeks	Amelioration of glycaemic control and induction of weight loss	STZ-induced diabetic male and female rats	[69]
	Succinate supplementation (5 mmol/l or 10 mmol/l) for 5 weeks	Beige adipose tissue development	Mice fed an HFD for 5 weeks	[70]
	Sodium succinate in drinking water (1%, 1.5% and 2%) for 4 weeks	Induction of thermogenesis in brown adipose tissue and amelioration of glucose tolerance	Male mice fed an HFD for 4 weeks	[71]
	Dietary supplementation with sodium succinate (5% wt/wt) for 21 days	Improved glucose homeostasis through activation of intestinal gluconeogenesis	Male mice fed an HFHF diet for 21 days	[14]
	Dietary supplementation with succinate (5% wt/wt) for 4 weeks	Reduced hyperglycaemia through activation of intestinal gluconeogenesis	*ob*/*ob* male mice	[18]
Dichotomous				
	0.75 mg/ml succinic acid in drinking water for 6 weeks	Increased fasting glucose and LDL-cholesterol without changes in BW but attenuated adiposity and increased respiratory rate	Male mice fed an HFD for 4.5 months	[74]
	Dietary supplementation with disodium succinate (0.05%, 0.10%, 0.15% and 0.2% wt/wt) for 4 weeks	Increased food intake, BW, body-fat content, whole-body protein and energy deposition^a^; elevated fat accumulation in the intestine and the liver but ameliorated glucose tolerance; altered succinylation patterns in the liver and the intestine and modified gut microbiota	Zebrafish fed a control-check diet (with 0% succinate) supplemented with soybean oil (60 g/kg of control diet)	[75]

^a^Increased energy deposition refers to an increased energy gain and reduced energy expenditure in the form of standard metabolic energy

BW, body weight; HFHF, high-fat high-fructose; STZ, streptozocin

It is worth noting that the use of disodium succinate in some of the above-mentioned articles may introduce potential confounding factors due to hypertonicity. However, Lund et al demonstrated that, unlike the observed effects with sodium lactate, the anti-obesogenic effect of succinate administration is independent of sodium [76]. Finally, succinic acid derivatives or succinate combinations with other drugs have shown the potential to improve cognitive and depressive symptoms related to diabetes in humans [77]. In summary, the available data suggest that the administration of succinate during the early stages of obesity may offer potential benefits in counteracting weight gain and disturbances in glucose homeostasis. However, as obesity progresses, succinate's efficacy appears to be compromised due to the development of resistance, likely stemming from elevated circulating succinate concentrations.

#### Microbiota modulation and other indirect strategies

In addition to direct succinate administration, various strategies focusing on microbiota modulation and other indirect approaches have been employed to influence intestinal succinate production and absorption (Table 2). However, only two interventions, a lifestyle modification study involving women with obesity [3] and the administration of the succinate-consuming bacteria *Odoribacter laneus* in murine models of obesity and diabetes [16], have assessed modulation of circulating succinate levels. In both instances, a reduction in blood succinate was observed. In the first study, this reduction was associated with weight loss and a decrease in the ratio of succinate producers:consumers within the gut microbiota [3]. In the second study, reduced blood succinate was linked to improved glucose control and reduced inflammation [16]. Conversely, studies following pre- and probiotic administration or faecal microbiota transplantation have predominantly examined succinate levels or production within the gut or faeces, where increases in succinate or succinate producers have generally been associated with protective effects [14, 17, 18, 78]. Specifically, the genus *Prevotella*, with a particular focus on *Prevotella copri* [14, 17] has been extensively investigated. Strategies to enhance *Prevotella* presence in the gut, including dietary interventions with fibre [17], the oral gavage of bacteria [14, 17] or faecal microbiota transplantation [17], have resulted in improvements in glucose homeostasis[14, 17]. Similarly, hemicellulose supplementation has demonstrated enhanced glucose tolerance, improved gut function and reduced systemic inflammation in *db*/*db* mice [78].

**Table 2:** Effects of succinate modulation through dietary interventions, prebiotics and probiotics

Outcome	Methods	Effects	Participants/model used	Reference
Protective effects associated with decreased circulating succinate				
	Weight-loss intervention (hypocaloric Mediterranean diet + physical exercise programme) for 3 months	Reduced circulating succinate and ratio of succinate producers:consumers which were associated with weight loss	Women with obesity	[3]
	Oral administration of *O. laneus* (1–5×10^8^ cfu) for 3 weeks, on alternate days	Decreased circulating succinate levels, improving glucose tolerance and reducing inflammation through SUCNR1 involvement	Male mice with DIO and *db*/*db* genotype	[16]
Protective effects associated with increased succinate in faeces or caecum				
	Dietary supplementation with hemicellulose (10% and 20% wt/wt) for 8 weeks	Increased succinate in faeces; amelioration of glucose tolerance, gut function and systemic inflammation	*db*/*db* male mice fed a diet with defined composition (~5% fibre content)	[78]
	Daily oral gavage of *P. copri* for 7 days	Elevated levels of caecal succinate; improved glucose tolerance and increased glycogen accumulation in the liver, without alteration of portal succinate levels; no effects with a high-fat low-dietary fibre diet	Male mice fed a standard chow diet (low fat, low protein and high dietary fibre) and mice fed a high-fat low-dietary fibre diet	[14, 17]
	Oral gavage of *P. distasonis* (2×10^8^ cfu) for 4 weeks (*ob/ob* mice) or 5 weeks (HFD-fed mice)	Increased succinate levels in the gut; reduced weight gain, hyperglycaemia and hepatic steatosis	Male *ob/ob* mice, and male mice fed an HFD for 14 weeks	[18]
Protective effects associated with increased succinate-producing bacteria				
	Oral gavage of *B. wexlerae* (5×10^8^ cfu in 0.5 ml solution) thrice/week for 10 weeks	Reduced BW gain and fat accumulation; improved insulin response to glucose and decreased diabetic indicators; reduced accumulation of proinflammatory macrophages in adipose tissue; increased succinate levels in mature adipose fraction from adipose tissue and in the liver; no detectable faecal levels of succinate	Male mice fed an HFD for 10 weeks	[19]
	Daily topical treatment of *L. plantarum* LC38 (10^9^ cfu/ml) until wound healing in the control group	Enhanced wound healing activity and accelerated wound closure after 14 days of wound induction	Male rats with alloxan monohydrate-induced T1D	[79]
Protective effects associated with decreased succinate in faeces or gut				
	Dietary supplementation with fermented rice bran (0.239% wt/wt) for 8 weeks	Decreased faecal succinate levels; weight loss and amelioration of HFD-induced obesity through regulation of microbiota and host metabolism	Female mice fed an HFD for 8 weeks	[80]

BW, body weight; cfu, colony-forming units; DIO, diet-induced obesity; T1D, type 1 diabetes

Administration of *Parabacteroides distasonis*, another succinate-producing bacterium, led to an increase in gut succinate concentration concurrent with reduced weight gain, improved blood glucose levels and mitigated hepatic steatosis in mouse models of genetic and diet-induced obesity [18]. Meanwhile, *Blautia wexlerae*, also a succinate producer, counteracted obesity and diabetes induced by an HFD, with succinate levels reported to increase primarily in adipose tissue and the liver [19]. Focusing on diabetes complications, daily topical application of the beneficial bacterium *Lactiplantibacillus plantarum* was found to produce succinate and expedite wound healing in rat models of type 1 diabetes [79]. It is crucial to emphasise, however, that none of these studies have conclusively established that the observed beneficial effects are solely attributable to succinate. In fact, it is evident that the involvement of other metabolites, which are also likely to be modulated with these interventions, cannot be ruled out. In contrast, increasing succinate consumers in the gut through faecal microbiota transplantation, or decreasing succinate in the gut through supplementation with fermented rice bran has also proven effective in ameliorating obese and diabetic phenotypes in mice [20, 80]. These findings align with results obtained in our study, where a probiotic intervention involving *O. laneus* revealed that the beneficial effects of reducing succinate levels were contingent on its signalling capacities through SUCNR1 [16]. This underscores the need for further research to elucidate whether modulating succinate production or consumption in the gut holds therapeutic promise. The outcome may depend on the amount of succinate that enters the circulation and reaches other tissues, influenced by changes in its production:consumption ratio, cross-feeding reactions and intestinal permeability.

## Conclusions and future directions

In sum, our knowledge of the role of the succinate–SUCNR1 system in health and disease continues to grow. Despite their importance in maintaining metabolic and immune balance, succinate and its receptor can also contribute to chronic diseases, complicating therapeutic strategies. This is especially true in diabetes, where disrupted succinate signalling plays a part in disease progression. Heightened succinate levels in people with diabetes and animal models of this disease hint at a relationship between succinate and insulin resistance, disturbed glucose metabolism and co-existing conditions. As our review outlines, circulating and faecal succinate emerge as potential clinical tools for diabetes prediction. Although tissue-specific determination of succinate could provide more clinical value, blood and faecal succinate are more easily accessible and measurable via non-invasive methods, providing a window into the metabolic disruptions linked to diabetes, including changes in mitochondrial function, oxidative stress and dysbiosis. These characteristics make succinate a potentially valuable biomarker for early detection and risk stratification in diabetes. However, there is still much to learn; standardised measurement methods and large-scale studies are needed to validate succinate's utility in predicting diabetes. Due to its duality in function and source, its interactions with other metabolic factors and contradictory effects on metabolic health require a comprehensive research approach. As a central molecule in diabetes research, succinate offers insights into the dichotomous outcomes of metabolic diseases. Thus, understanding succinate's roles and interactions with other cellular pathways could be helpful for diabetes management. Moreover, striking a balance between blocking the harmful effects of SUCNR1 while maintaining its beneficial ones offers a promising path for novel diabetes treatments. We must fully uncover the mechanisms driving succinate–SUCNR1 signalling and their impact on disease progression; this knowledge could help to develop interventions to curb succinate’s detrimental effects in diabetes, improving patient outcomes.

### Supplementary Information

Below is the link to the electronic supplementary material.

Supplementary file1 (PPTX 245 KB)

## Abbreviations

COX: Cyclooxygenase
GDM: Gestational diabetes mellitus
GPCR: G-protein-coupled receptor
HFD: High-fat diet
NAFLD: Non-alcoholic fatty liver disease
NASH: Non-alcoholic steatohepatitis
SCFA: Short-chain fatty acid
SDH: Succinate dehydrogenase
SUCNR1: Succinate receptor 1
VEGF: Vascular endothelial growth factor
