# Supplementary material for: Type 2 diabetes and succinate: unmasking an age-old molecule
Source: Diabetologia. 2024 Jan 5;67(3):430–42. doi: 10.1007/s00125-023-06063-7 (PMC10844351; doi:10.1007/s00125-023-06063-7)
Supplement: Supplementary file 1 — Supplementary file1 (PPTX 245 KB) [file 125_2023_6063_MOESM1_ESM.pptx]

## Slide 1
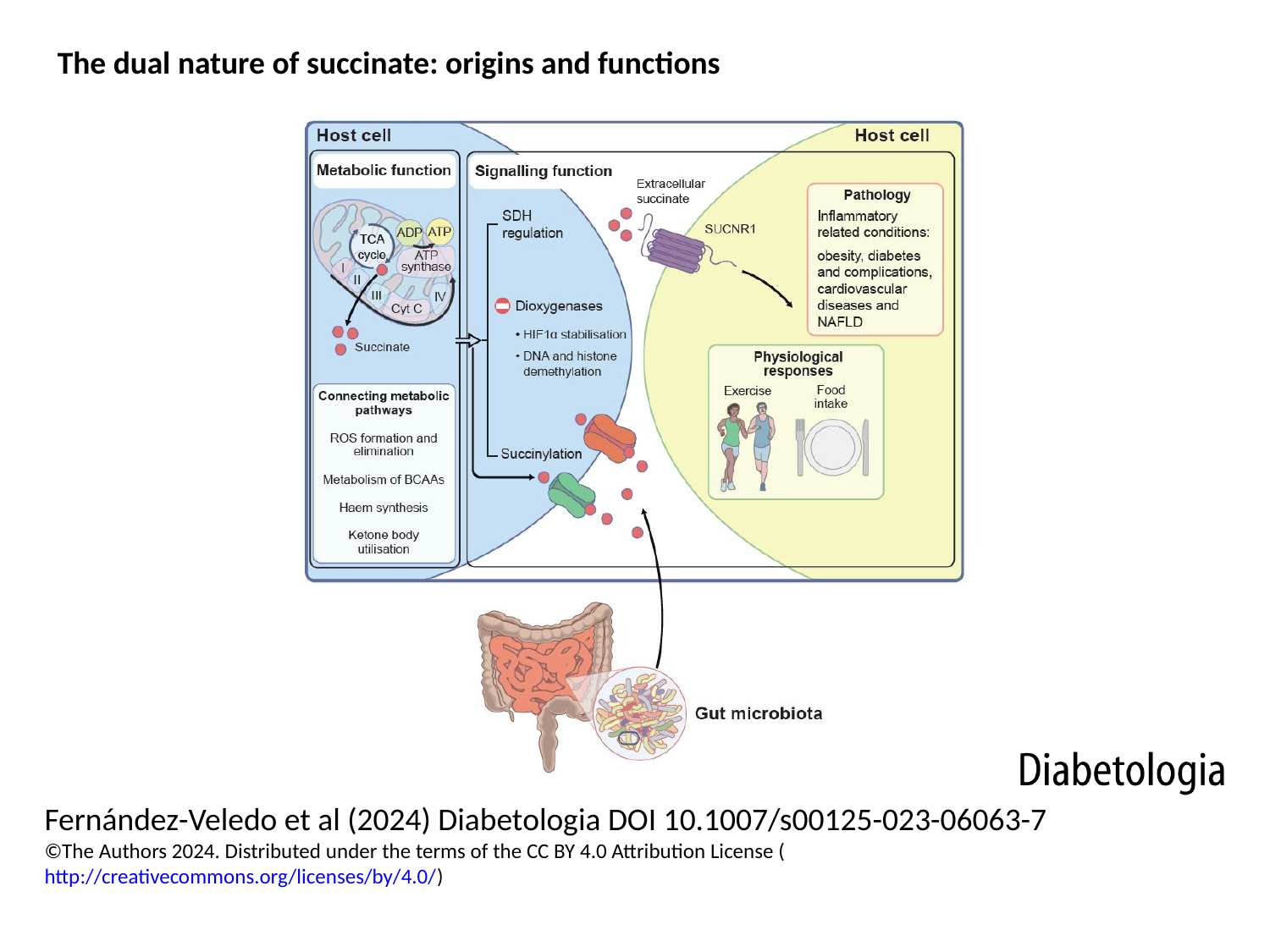

The dual nature of succinate: origins and functions
Fernández-Veledo et al (2024) Diabetologia DOI 10.1007/s00125-023-06063-7
©The Authors 2024. Distributed under the terms of the CC BY 4.0 Attribution License (http://creativecommons.org/licenses/by/4.0/)
